# The Relative Contributions of Facial Shape and Surface Information to Perceptions of Attractiveness and Dominance

**DOI:** 10.1371/journal.pone.0104415

**Published:** 2014-10-28

**Authors:** Jaimie S. Torrance, Joanna Wincenciak, Amanda C. Hahn, Lisa M. DeBruine, Benedict C. Jones

**Affiliations:** Institute of Neuroscience & Psychology, University of Glasgow, Glasgow, United Kingdom; University of Goettingen, Germany

## Abstract

Although many studies have investigated the facial characteristics that influence perceptions of others’ attractiveness and dominance, the majority of these studies have focused on either the effects of shape information or surface information alone. Consequently, the relative contributions of facial shape and surface characteristics to attractiveness and dominance perceptions are unclear. To address this issue, we investigated the relationships between ratings of original versions of faces and ratings of versions in which either surface information had been standardized (i.e., *shape-only* versions) or shape information had been standardized (i.e., *surface-only* versions). For attractiveness and dominance judgments of both male and female faces, ratings of *shape-only* and *surface-only* versions independently predicted ratings of the original versions of faces. The correlations between ratings of original and *shape-only* versions and between ratings of original and *surface-only* versions differed only in two instances. For male attractiveness, ratings of original versions were more strongly related to ratings of *surface-only* than *shape-only* versions, suggesting that surface information is particularly important for men’s facial attractiveness. The opposite was true for female physical dominance, suggesting that shape information is particularly important for women’s facial physical dominance. In summary, our results indicate that both facial shape and surface information contribute to judgments of others’ attractiveness and dominance, suggesting that it may be important to consider both sources of information in research on these topics.

## Introduction

Judgments of others’ facial attractiveness and dominance play an important role in social perceptions and have a significant influence on social interactions [1]–[3]. For example, people prefer to date, associate with, and employ attractive individuals [1], [2]. By contrast, people tend to avoid cooperating with individuals displaying facial cues of dominance, potentially because dominant individuals are more likely to exploit others’ trust [4]. This effect of dominance on cooperation can be modulated by the salience of in-group versus out-group competition for resources, however [5].

Many studies examining the specific characteristics that influence judgments of others’ facial attractiveness and dominance have investigated the effects of aspects of face shape. For example, exaggerating sex-typical shape characteristics in images of women’s faces increases their perceived attractiveness [6], [7], but tends to decrease their perceived dominance [6], [8]. By contrast, although exaggerating sex-typical shape characteristics in images of men’s faces reliably increases their perceived dominance [6], [8], the effect on men's attractiveness is variable [9]. Experimentally manipulating some shape characteristics in face images, such as symmetry [10], [11] and prototypicality [12], [13], [14], affects their attractiveness, while experimentally manipulating other shape characteristics, such as facial width, affects perceptions of dominance-related traits, such as aggression [15]. Together these results demonstrate that shape information in faces influences perceptions of both others’ attractiveness and others’ dominance.

While many studies have investigated the effects of face shape characteristics on attractiveness and dominance perceptions, fewer studies have examined the effects of facial surface information on these judgements. Nonetheless, yellower, lighter, and more homogenous facial skin is considered attractive [16]–[18] and increasing red coloration in face images increases their perceived dominance [19]. Other aspects of facial surface information, such as men’s facial hair [20], also influence dominance judgments. Similarly, aspects of facial surface information, including prototypicality [14], the luminance contrast between different regions of the face [21], [22], and facial hair [23], [24], have been shown to influence judgments of facial attractiveness. Together, these results suggest that facial surface information can also influence perceptions of attractiveness and dominance.

Despite the relatively large number of studies investigating the specific characteristics that influence perceptions of facial attractiveness and dominance, the *relative* contributions of shape and surface information to these judgements are unclear. For example, studies that have tested for independent effects of shape and surface information on facial attractiveness or dominance judgments have typically (i) manipulated either shape [6]–[8] or surface [19] characteristics only, (ii) independently manipulated both shape and surface characteristics [12], [14], or (iii) compared the correlations between ratings and specific, individual shape or surface characteristics of faces [18]. Importantly, these approaches cannot clarify the relative contribution of facial shape and surface characteristics to attractiveness and dominance judgments, other than by demonstrating that some specific, individual shape and surface characteristics have independent effects on attractiveness and dominance judgments. That the relative contribution of facial shape and surface characteristics to attractiveness and dominance judgments of faces has been the focus of so little empirical investigation is perhaps surprising, given several studies have previously investigated this issue in relation to judgments of others’ race, sex, and identity from facial cues (e.g., [25]–[27]).

In light of the above, the current study directly assessed the relative contribution of facial shape and surface characteristics to attractiveness and dominance judgments. Participants rated the attractiveness or dominance of either original versions of faces (original face condition), versions in which surface information was standardized by warping the average face for the sample into the shape of each original face (*shape-only* face condition), or versions in which shape information was standardized by warping each original face into the average shape for the sample (*surface-only* face condition). For each judgment, we then (i) tested whether ratings of the *shape-only* and *surface-only* versions independently predicted ratings of the original faces and (ii) compared the correlations between ratings of the original and *shape-only* versions and between ratings of the original and *surface-only* versions. These latter tests directly assess the relative contribution of perceptions of facial shape and surface information for attractiveness and dominance judgments.

Since some research [28]–[30] has distinguished between perceptions of physical dominance (i.e., dominance perceptions that focus on cues of physical formidability) and social dominance (i.e., dominance perceptions that focus on cues of social status), each type of face (original, *shape-only*, *surface-only*) was also rated for physical dominance and social dominance. Collecting these data allowed us to test whether ratings of social and physical dominance are more strongly correlated in men than in women, as some research has suggested [29], [30].

## Methods

### Stimuli

First, we took face photographs of 50 white men (mean age = 20.79 years, SD = 2.41 years) and 50 white women (mean age = 21.79 years, SD = 3.62 years) under standardized lighting conditions and against a constant background. All individuals posed front on to the camera with neutral expressions and direct gaze. Following other recent studies investigating the role of surface information in face perceptions [31], these images were then color-calibrated using a procedure described in Hong et al. [32]. None of the women who were photographed were wearing makeup and none of the men who were photographed had full beards. Images were aligned on pupil position. Photographs were taken with a Nikon D300s and Interfit Super Cool-Lite 5 lights. Color calibration was carried out using a GretagMacbeth 24 square color calibration chart.

**Figure 1:**
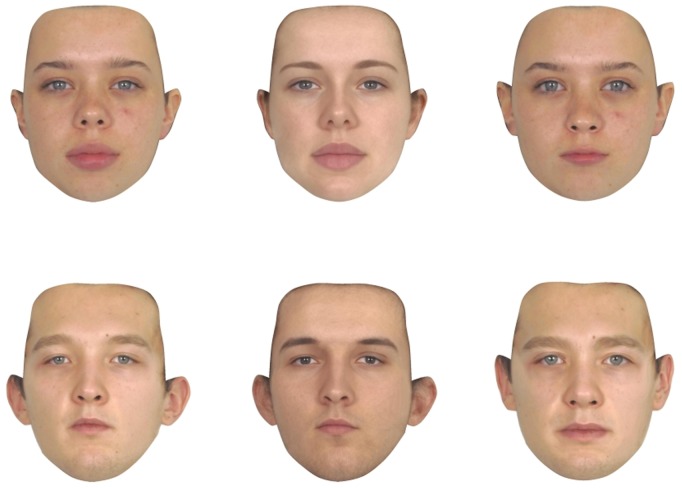
Examples of original (left), *shape-only* (centre), and *surface-only* (right) versions of female (top row) and male (bottom row) faces used in the study. *Shape-only* versions were manufactured by warping a prototype with the average color and texture information for the sample into the shape of each individual face. *Surface-only* versions were manufactured by warping each individual face into the average shape for the entire sample.

Following Little and Hancock [12], we then manufactured versions of each of the individual face images that had standardized surface information (*shape-only* images) and standardized 2D shape information (*surface-only* images). Example stimuli are shown in Figure 1. To create these *shape-only* and *surface-only* images, we first created a male prototype face with the average shape, color, and texture information for the set of 50 male face images and a female prototype face with the average shape, colour, and texture information for the set of 50 female face images. These prototypes were created with computer graphic techniques widely used to manufacture prototype face stimuli for psychological testing [33].

To create *shape-only* versions of each of the 50 male faces, the male prototype was warped into the shape of each of the individual male faces. Similarly, *shape-only* versions of each of the 50 female faces were manufactured by warping the female prototype into the shape of each of the individual female faces. To create *surface-only* versions of each of the 50 male faces, each of the individual male faces was warped into the shape of the male prototype. *Surface-only* versions of each of the 50 female faces were also manufactured by warping each of the individual female faces into the shape of the female prototype. Details of the computer graphic techniques used to perform these image manipulations are given in Tiddeman et al. [33]. Finally, all faces images were masked so that hairstyle and clothing were not visible.

### Procedure

Six hundred and forty-five women (mean age = 22.36 years, SD = 4.95 years) and 265 men (mean age = 24.70 years, SD = 5.91 years) took part in the study, which was run online. Participants were recruited by following links to an online face perception study on social bookmarking sites, such as stumbleupon.com. Previous research has demonstrated that responses to shape and surface information in faces in online and laboratory studies are similar [34], [35]. Each participant was randomly allocated to one condition in which they rated either the original versions of male or female faces, the *shape-only* versions of male or female faces, or the *surface-only* versions of male or female faces for either attractiveness, dominance, social dominance, or physical dominance. Participants were randomly allocated to only one condition, rather than rating the same individuals in multiple conditions, because the latter method may inflate the correlations between ratings of faces made in different conditions [2].

Definitions of social and physical dominance were adapted from those used in previous studies [28], [30]. Socially dominant individuals were defined as “those who are more likely to tell other people what to do, are respected, influential, and often leaders”. Physically dominant individuals were defined as “those who would probably win if they got in a fistfight with a person of the same sex and age”. Following other studies in which faces were rated for attractiveness or dominance [6],[19], these traits were not defined. Attractiveness, dominance, social dominance, and physical dominance were rated using 1 (much less attractive/dominant/socially dominant/physically dominant than average) to 7 (much more attractive/dominant/socially dominant/physically dominant than average) scales. Trial order was randomized between participants.

Thirty-one of our participants (24 women, 7 men), none of whom provided any of the ratings described above, rated the original versions of the male faces in a randomized order for the amount of stubble using a 1 (much less than average) to 7 (much more than average scale). Although none of the men photographed for our study had full beards, we collected these stubble ratings so that we could control for the possible effects of facial hair on social judgments in our analyses of ratings of male faces. This part of the study was also run online.

This research was approved by the Psychology Ethics Committee University of Glasgow. In this online study, participants provided their informed consent by clicking on the “I consent” button in response to the question “Do you consent to participate in this study?”.

### Initial processing of data

We calculated the mean attractiveness ratings for each of the original versions of the male and female faces, each of the *shape-only* versions of the male and female faces, and each of the *surface-only* versions of the male and female faces. Corresponding mean ratings were also calculated for dominance, social dominance, and physical dominance judgments (correlations between men’s and women’s ratings are given in the supplemental materials file Correlations S1). Means and SDs for these ratings, together with measures of inter-rater agreement (Cronbach’s alphas), are shown in Table 1. These mean ratings were used in our main analyses. Dominance ratings where the type of dominance was unspecified (i.e., not ratings from the social or physical dominance conditions) are referred to hereon as “general dominance”. Inter-rater agreement was high (all Cronbach’s alphas>.72) for each combination of trait (attractiveness, general dominance, social dominance, physical dominance), sex of face (male, female), and condition (original, *shape-only*, *surface-only*), except for physical dominance ratings of *surface-only* versions of female faces, for which inter-rater agreement was lower (Cronbach’s alpha = .52). Mean ratings of men’s stubble were also calculated (M = 2.85, SD = .90). Inter-rater agreement for these ratings was high (Cronbach’s alpha = .96). Kolmogorov-Smirnov tests showed that all variables were normally distributed (all p>.14). The data file containing analyzed scores is given in our supplemental materials file Data S1.

**Table 1:** Means and SDs for ratings of the attractiveness, general dominance, social dominance, and physical dominance of original, shape-only, and surface-only versions of male and female faces.

Trait	Sex of face	Condition	N (raters)	Mean	SD	Cronbach’s alpha
attractiveness	male	original	39	2.42	0.43	.88
attractiveness	female	original	37	3.01	0.74	.95
general dominance	male	original	37	3.45	0.64	.89
general dominance	female	original	35	3.47	0.45	.76
social dominance	male	original	35	3.30	0.63	.89
social dominance	female	original	37	3.26	0.56	.88
physical dominance	male	original	37	3.23	0.62	.90
physical dominance	female	original	38	3.54	0.42	.75
attractiveness	male	shape-only	35	2.73	0.59	.91
attractiveness	female	shape-only	35	2.97	0.56	.90
general dominance	male	shape-only	35	3.66	0.43	.80
general dominance	female	shape-only	36	3.85	0.46	.78
social dominance	male	shape-only	37	3.62	0.42	.76
social dominance	female	shape-only	37	3.64	0.44	.75
physical dominance	male	shape-only	37	3.62	0.39	.73
physical dominance	female	shape-only	37	3.55	0.43	.78
attractiveness	male	surface-only	39	2.96	0.60	.92
attractiveness	female	surface-only	37	3.47	0.53	.91
general dominance	male	surface-only	35	3.88	0.54	.87
general dominance	female	surface-only	40	3.73	0.37	.74
social dominance	male	surface-only	37	3.62	0.59	.88
social dominance	female	surface-only	35	3.63	0.55	.82
physical dominance	male	surface-only	37	3.77	0.59	.89
physical dominance	female	surface-only	35	3.60	0.31	.52

Inter-rater agreement (Cronbach’s alpha) is also given.

## Results

First, we investigated whether ratings of *shape-only* or *surface-only* versions of faces were the better predictor of ratings of the attractiveness, general dominance, social dominance, and physical dominance of *original* versions of faces. We did this by first calculating the correlations between ratings of *original* and *shape-only* versions and between ratings of *original* and *surface-only* versions and then comparing these correlations (greater correlation versus weaker correlation) using Steiger’s test [36]. Steiger’s test was developed specifically for comparing correlations from a correlation matrix and allows the correlation between variable A and variable B to be compared with the correlation between variable A and variable C, taking into account the strength of the correlation between variable B and variable C [36]. Separate tests were carried out for each combination of trait (attractiveness, general dominance, social dominance, physical dominance) and sex of face (male, female). Results are summarized in Table 2. The correlations between ratings of *shape-only* and *surface-only* versions of faces were not significant for any combination of sex of face and trait (all absolute r<.25, all p>.09).

**Table 2:** Simple correlations between ratings of original and shape-only versions and between ratings of original and surface-only versions of male and female faces.

Trait	Sex of face	Correlation between ratings of original and shape-only versions		Correlation between ratings of original and surface-only versions		Results of Steiger’s test	
		*r*	*p*	*r*	*p*	*z*	*p*
attractiveness	male	.41	.003	.72	<.001	2.21	.027
attractiveness	female	.71	<.001	.72	<.001	0.10	.918
general dominance	male	.56	<.001	.73	<.001	1.45	.147
general dominance	female	.47	.001	.36	.011	0.59	.557
social dominance	male	.50	<.001	.68	<.001	1.32	.187
social dominance	female	.42	.002	.51	<.001	0.52	.605
physical dominance	male	.57	<.001	.65	<.001	0.63	.532
physical dominance	female	.78	<.001	.18	.209	3.89	<.001

Results of tests for significant differences between these correlations (greater correlation versus weaker correlation) are also shown. N = 50 for all tests.

Correlations between ratings of *original* and *shape-only* versions and between ratings of *original* and *surface-only* versions were significant in all cases (all r>.36, all N = 50, all p<.012), *except* the correlation between ratings of the physical dominance of *original* and *surface-only* versions of female faces, which was not significant (r = .18, N = 50, p = .209). The Steiger’s tests showed no significant differences for any of the comparisons (all z<1.46, all p>.145), with two exceptions. First, the correlation between attractiveness ratings of *original* and *surface-only* versions of men’s faces was significantly stronger than the correlation between attractiveness ratings of *original* and *shape-only* versions of men’s faces (z = 2.21, p = .027). Second, the correlation between physical dominance ratings of *original* and *shape-only* versions of women’s faces was significantly stronger than the correlation between physical dominance ratings of *original* and *surface-only* versions of women’s faces (z = 3.89, p<.001).

Next, we tested whether ratings of *shape-only* and *surface-only* versions of faces both independently predicted ratings of the attractiveness, general dominance, social dominance, and physical dominance of *original* versions of faces. Separate regression analyses, in which ratings of *original* versions were entered as the dependent variable and ratings of *shape-only* and *surface-only* versions were entered simultaneously as predictors, were carried out for each combination of trait (attractiveness, general dominance, social dominance, physical dominance) and sex of face (male, female). Results are summarized in Table 3. Significant, independent positive relationships between ratings of *shape-only* and *original* versions and between ratings of *surface-only* and *original* versions were seen in each analysis.

**Table 3:** Summary results of analyses testing for independent contributions of ratings of shape-only and surface-only versions of male and female faces.

Trait	Sex of face	Ratings of shape-only versions			Ratings of surface-only versions		
		*t*	standardized beta	*p*	*t*	standardized beta	*p*
attractiveness	male	4.19	.36	<.001	8.05	.70	<.001
attractiveness	female	9.13	.57	<.001	9.36	.58	<.001
general dominance	male	5.33	.43	<.001	7.91	.64	<.001
general dominance	female	4.63	.53	<.001	3.72	.42	.001
social dominance	male	5.16	.45	<.001	7.37	.64	<.001
social dominance	female	4.30	.46	<.001	5.04	.54	<.001
physical dominance	male	5.11	.47	<.001	6.14	.56	<.001
physical dominance	female	9.20	.79	<.001	2.47	.21	.017

Repeating these analyses of ratings of male faces, this time with ratings of men’s stubble included as an additional predictor, did not alter the pattern of results shown in Table 3. Independent positive relationships between ratings of *shape-only* and *original* versions of men’s faces and between ratings of *surface-only* and *original* versions of men’s faces were seen for each trait. Additionally, ratings of men’s stubble did not predict ratings of the *original* versions in any of these analyses (all absolute t<0.97, all absolute standardized beta<0.10, all p>.34). Together, these additional tests suggest that the effect of stubble on perceptions of men’s faces contributed very little to the observed effects of surface information on perceptions of men’s faces in this sample.

Previous research has suggested that ratings of men’s social and physical dominance may be correlated more strongly than ratings of women’s social and physical dominance (e.g., [29], [30]). To investigate this issue, we compared the correlations between ratings of men’s social and physical dominance with the correlations between ratings of women’s social and physical dominance. These comparisons used the Fisher r-to-z transformation and were carried out separately for ratings of *original*, *surface-only*, and *shape-only* versions of faces. For ratings of *original* versions, social and physical dominance ratings were positively correlated for male (r = .85, N = 50, p<.001), but not female (r = .07, N = 50, p = .61), faces, and these correlations were significantly different from one another (z = 5.75, p<.001). For ratings of *surface-only* versions, social and physical dominance ratings were positively correlated for male (r = .72, N = 50, p<.001), but not female (r = .26, N = 50, p = .070), faces, and these correlations were significantly different from one another (z = 3.11, p = .002). For ratings of *shape-only* versions, social and physical dominance ratings were positively correlated for both male (r = .45, N = 50, p = .001) and female (r = .37, N = 50, p = .009) faces, and these correlations were not significantly different from one another (z = 0.47, p = .64). For ratings of *original* versions of faces, general dominance was predicted equally well by social and physical dominance ratings (male faces: z = 0.73, p = .47; female faces: z = 0.15, p = .88).

## Discussion

For each type of judgment (i.e., ratings of attractiveness, general dominance, social dominance, and physical dominance) we found that ratings of original versions of both male and female faces were independently predicted by ratings of *shape-only* versions and ratings of *surface-only* versions of faces. These results are summarized in Table 3 and are consistent with previous research in which independently manipulating shape [6], [8] and surface characteristics [19], [21] in faces altered perceptions of their attractiveness and dominance.

In addition to finding that attractiveness and dominance judgments of faces were independently predicted by ratings of *shape-only* versions and ratings of *surface-only* versions, we also found that the correlations between ratings of original and *surface-only* versions of faces and between ratings of original and *shape-only* versions of faces were generally similar (i.e., were generally not significantly different from one another). These results are summarized in Table 2. There were two exceptions to this pattern of results, however.

First, the correlation between attractiveness ratings of original and *surface-only* versions of men’s faces was significantly stronger than the correlation between attractiveness ratings of original and *shape-only* versions of men’s faces, suggesting that surface information is more important than shape information when judging the attractiveness of men’s faces. This pattern of results complements recent work suggesting that measurements of facial coloration predict ratings of men’s facial attractiveness better than do measurements of sexually dimorphic aspects of face shape [18]. However, our results extend this finding by showing that this pattern of results occurs more generally for comparisons of the importance of shape and surface information in men’s faces (i.e., is not specific to the comparison of facial coloration and sexually dimorphic shape characteristics only).

Second, the correlation between physical dominance ratings of original and *shape-only* versions of women’s faces was significantly stronger than the correlation between physical dominance ratings of original and *surface-only* versions of women’s faces, suggesting that shape information is more important for judgments of the physical dominance of women’s faces than is surface information. Indeed, the relatively low inter-rater agreement for physical dominance judgments of *surface-only* versions of women’s faces is consistent with people not routinely using surface information to assess women’s physical dominance.

Some research has suggested that correlations between ratings of social and physical dominance are stronger for ratings of men’s faces than women’s faces [29], [30]. Support for this proposal from our analyses was mixed, however. Correlations between ratings of physical and social dominance were significantly stronger for male faces than female faces when we analyzed ratings of original and *surface-only* versions. By contrast, the correlations between ratings of social and physical dominance were not significantly stronger for male faces than female faces when we analyzed ratings of *shape-only* versions. This pattern of results was unexpected and suggests that the proposed sex difference in the relationships between physical and social dominance may be more complicated than some researchers have suggested.

Our analyses suggest that shape and surface information in faces are potentially important cues for judgments of others’ attractiveness and dominance. However, our study does not address the relative contributions of *specific* shape and surface characteristics to social judgments of faces. Candidate surface characteristics include information about skin texture, color, contrast, and luminance. Candidate shape characteristics include information about prototypicality, adiposity, and demeanour. Further work is needed to explore these issues. Additional analyses of ratings of men’s faces, in which we controlled for the possible effects of stubble on judgments of men’s faces, showed that the effects of surface information on perceptions of men’s faces in our study were unlikely to be solely due to previously reported effects of facial hair on perceptions of men’s faces [20], [23], [24]. Indeed, although we found no links between judgments of men’s faces and ratings of their stubble, facial hair may make a more substantial contribution to judgments of men’s faces in samples where there was a wider range of facial hair represented and/or in which some men displayed full beards. Again, further research is needed to explore this issue.

In summary, we found that shape and surface information in faces independently contributed to attractiveness and dominance judgments of both men’s and women’s faces. Moreover, we found that the correlations between ratings of *shape-only* and original versions of faces and between ratings of *surface-only* and original versions of faces were generally very similar, although surface information appeared to be more important for men’s (but not women’s) attractiveness than shape information. The reasons for the sex difference in this latter effect are unclear, although it is consistent with research suggesting that sexually dimorphic shape information in faces [2] and cues of adiposity [37] have weaker effects on men’s than women’s facial attractiveness. Collectively, our data demonstrate that both shape and surface information in faces are important for social perceptions of faces, underlining the importance of studying both types of information to understand the facial cues that influence attractiveness and dominance judgments.

## Supporting Information

Correlations S1 (DOC)

Data S1 (XLS)
