# Supplementary material for: The Relative Contributions of Facial Shape and Surface Information to Perceptions of Attractiveness and Dominance
Source: PLoS One. 2014 Oct 28;9(10):e104415. doi: 10.1371/journal.pone.0104415 (PMC4211661; doi:10.1371/journal.pone.0104415)
Supplement: Correlations S1 — (DOC) [file pone.0104415.s001.doc]

**Supplemental Materials (Torrance et al.)**

Correlations between men’s and women’s ratings for each combination of face sex (male, female), condition (shape-only, surface-only, original), trait (attractiveness, dominance, social dominance, physical dominance).

| **Trait** | **Sex of face** | **Condition** | **Correlation (r)** |
| --- | --- | --- | --- |
| attractiveness | male | original | .80 |
| attractiveness | female | original | .84 |
| general dominance | male | original | .77 |
| general dominance | female | original | .45 |
| social dominance | male | original | .77 |
| social dominance | female | original | .70 |
| physical dominance | male | original | .80 |
| physical dominance | female | original | .51 |
| attractiveness | male | shape-only | .81 |
| attractiveness | female | shape-only | .74 |
| general dominance | male | shape-only | .66 |
| general dominance | female | shape-only | .49 |
| social dominance | male | shape-only | .59 |
| social dominance | female | shape-only | .78 |
| physical dominance | male | shape-only | .49 |
| physical dominance | female | shape-only | .72 |
| attractiveness | male | surface-only | .84 |
| attractiveness | female | surface-only | .86 |
| general dominance | male | surface-only | .78 |
| general dominance | female | surface-only | .45 |
| social dominance | male | surface-only | .76 |
| social dominance | female | surface-only | .59 |
| physical dominance | male | surface-only | .77 |
| physical dominance | female | surface-only | .32 |
